# Small cell neuroendocrine carcinoma and poorly differentiated rhabdomyosarcomas of the urinary bladder in adults—A comparative analysis in favor of a common histogenesis

**DOI:** 10.1007/s00428-024-03835-3

**Published:** 2024-06-04

**Authors:** Veronika Bahlinger, Robert Stoehr, Arndt Hartmann, Ondřej Hes, Abbas Agaimy

**Affiliations:** 1 Institute of Pathology, Friedrich-Alexander University Erlangen-Nürnberg (FAU), University Hospital Erlangen (UKER), Erlangen, Germany; 2 Comprehensive Cancer Center Erlangen-EMN (CCC ER-EMN), Erlangen, Germany; 3 Department of Pathology, Charles University, Medical Faculty and Charles University Hospital Plzen, Plzen, Czech Republic; 4 Present Address: Department of Pathology and Neuropathology, University Hospital and Comprehensive Cancer Center Tubingen, Tubingen, Germany

**Keywords:** Small cell neuroendocrine carcinoma, Rhabdomyosarcoma, Urinary bladder, *TERT* promoter mutations, *FOXO1* gene fusions

## Abstract

Rhabdomyosarcoma (RMS) of the urinary bladder in adults and elderly is an exceptionally rare neoplasm that displays poorly differentiated solid (alveolar-like) small cell pattern, frequently indistinguishable from small cell neuroendocrine carcinoma (SCNEC). However, the histogenesis of RMS and SCNEC and their inter-relationship have not been well studied and remained controversial. We herein analyzed 23 SCNEC and 3 small round cell RMS of the bladder for neuroendocrine (synaptophysin + chromogranin A) and myogenic (desmin + myogenin) marker expression and for *TERT* promoter mutations. In addition, the RMS cohort and one SCNEC that was revised to RMS were tested for gene fusions using targeted RNA sequencing (TruSight Illumina Panel which includes FOXO1 and most of RMS-related other genes). Overall, significant expression of myogenin and desmin was observed in one of 23 original SCNEC justifying a revised diagnosis to RMS. On the other hand, diffuse expression of synaptophysin was noted in 2 of the 4 RMS, but chromogranin A was not expressed in 3 RMS tested. *TERT* promoter mutations were detected in 15 of 22 (68%) SCNEC and in two of three (67%) assessable RMS cases, respectively. None of the four RMS cases had gene fusions. Our data highlights phenotypic and genetic overlap between SCNEC and RMS of the urinary bladder. High frequency of *TERT* promoter mutations in SCNEC is in line with their presumable urothelial origin. In addition, the presence of *TERT* promoter mutation in 2 of 3 RMS and lack of *FOXO1* and other gene fusions in all 4 RMSs suggest a mucosal (urothelial) origin, probably representing extensive monomorphic rhabdomyoblastic transdifferentiation in SCNEC.

## Introduction

Urothelial bladder cancer is the fifth most common cancer in men worldwide and one of the most cost consuming malignancies [1]. Divergent histomorphological differentiation accompanied by distinct clinical outcomes is a main characteristic of bladder cancer. Some urothelial carcinoma subtypes are associated with poor clinical outcome, but histology-tailored therapeutic recommendations are not available yet [2]. Among the reported bladder carcinoma subtypes, neuroendocrine cancer is a rare and clinically aggressive subtype accounting for < 1% of bladder tumors [3]. Small cell neuroendocrine carcinoma (SCNEC) of the urinary bladder is the major representative in the spectrum of neuroendocrine bladder cancer, but mixed types do occur [4]. From a clinical point of view, SCNEC is characterized by highly aggressive course heralded by early metastasis and worse prognosis with 80% of patients dying within 5 years after diagnosis [5].

Skeletal muscle differentiation occurs rarely across a variety of human malignancies. However, it has a greater tendency to occur among neural/neuroendocrine neoplasms [6]. Moreover, rhabdomyosarcoma (RMS) of the bladder in adults and elderly frequently shows solid small round cell pattern recapitulating poorly differentiated solid alveolar RMS of other sites. This pattern closely resembles SCNEC [7]. Nevertheless, clinical prognosis as well as therapeutic implications as for example choice of chemotherapy differs significantly for the two entities [3, 8].

The aim of this study was to compare SCNEC and adult-onset RMS of the bladder for skeletal muscle differentiation and neuroendocrine marker expression, respectively. In addition, we tested both cohorts for *TERT* promoter gene mutations (as surrogate for urothelial origin) and the RMS cohort for *FOXO1* fusions (as marker for majority of alveolar RMS cases) using a large, targeted RNA fusion detection panel to assess the hypothesis, if both entities represent two phenotypic patterns of same histogenetic disease.

## Materials and methods

### Study cohort

Twenty-three cases of SCNEC and three RMS cases were retrieved from routine and consultation files of our departments. Histological reassessment was done in keeping with the currently valid version of the World Health Organization (WHO) 2022 classification for bladder tumors [9]. For each tumor, transurethral resection specimens were used for analysis.

### Immunohistochemical analysis

Immunohistochemical analysis (IHC) was performed using a Ventana BenchMark Ultra autostainer (Ventana Medical Systems Inc, 1910 Innovation Park Drive, Tucson, Arizona, USA) according to the manufacturer’s instructions. Whole tissue consecutive, 3-µm cuts were made from embedded tissues. The following antibodies were used for analysis: desmin (D33, mouse monoclonal, Dako, dilution 1:50), myogenin (F5D, monoclonal mouse, Dako, dilution 1:50), synaptophysin (rabbit polyclonal, ThermoScientific, dilution 1:350), and chromogranin A (DAK-A3, monoclonal mouse, Dako, 1:400). Expression of these four markers was scored as strong, moderate or weak and diffuse or focal. Diverse other markers were used in a case-to-case basis according to the most pertinent differential diagnostic considerations at time of initial biopsy assessment.

### DNA isolation

The manual microdissection of tumor tissue was performed carefully after previous annotation of the SCNEC as well as RMS area on a hematoxylin and eosin (H&E)-stained slide. DNA isolation was performed using the Maxwell16® LEV Blood DNA Kit (Promega, Mannheim, Germany) according to the manufacturer’s instructions.

### TERT promoter analysis

The mutation analysis of the *TERT* promoter was performed as described elsewhere using SNaPshot analysis of the *TERT* core promoter with an ABI Prism 3500 Genetic Analyzer and the SNaPshot-Multiplex-Kit (Applied Biosystems, Foster City, CA, USA) according to the manufacturer’s instructions [10]. SNaPshot assays were designed to detect the three-hotspot mutations at positions − 146, − 124, and − 57 bp of the *TERT* promoter.

### RNA isolation

For all RMS samples and one SCNEC with diffuse rhabdomyogenic features, RNA was isolated from formalin-fixed paraffin embedded (FFPE) tissue sections using RNeasy FFPE Kit (Qiagen, Hilden, Germany) and quantified spectrophotometrically using NanoDrop-1000 (Thermo Fisher Scientific, Waltham, MA, USA).

### RNA fusion analysis

For all RMS samples and one SCNEC with myogenic features, molecular analysis was performed using the TruSight RNA Fusion panel (Illumina, Inc., San Diego, CA, USA) with 500 ng RNA as input according to the manufacturer’s protocol. Libraries were sequenced on a MiSeq system system (Illumina, Inc., San Diego, CA, USA) with > 3 million reads per case, and sequences were analyzed using the RNA-Seq Alignment workflow, version 2.0.1 (Illumina, Inc., San Diego, CA, USA). The Integrative Genomics Viewer (IGV), version 2.2.13 13 (Broad Institute, University of California, CA, USA) was used for data visualization [11].

### FOXO1 FISH translocation analysis

In two RMS cases (revised SCNEC case 14 and RMS case 1) the Zyto*Light*®SPEC FOXO1 Dual Color Break Apart Probe (ZytoVision GmbH, Bremerhaven, Germany) was used to detect translocations involving the chromosomal region 13q14.11 harboring the *FOXO1* gene and fluorescence in situ hybridization (FISH) study was performed following the manufacturer’s recommendations.

## Results

### Clinical characteristics and histological review of the SCNEC cohort

**Fig. 1:**
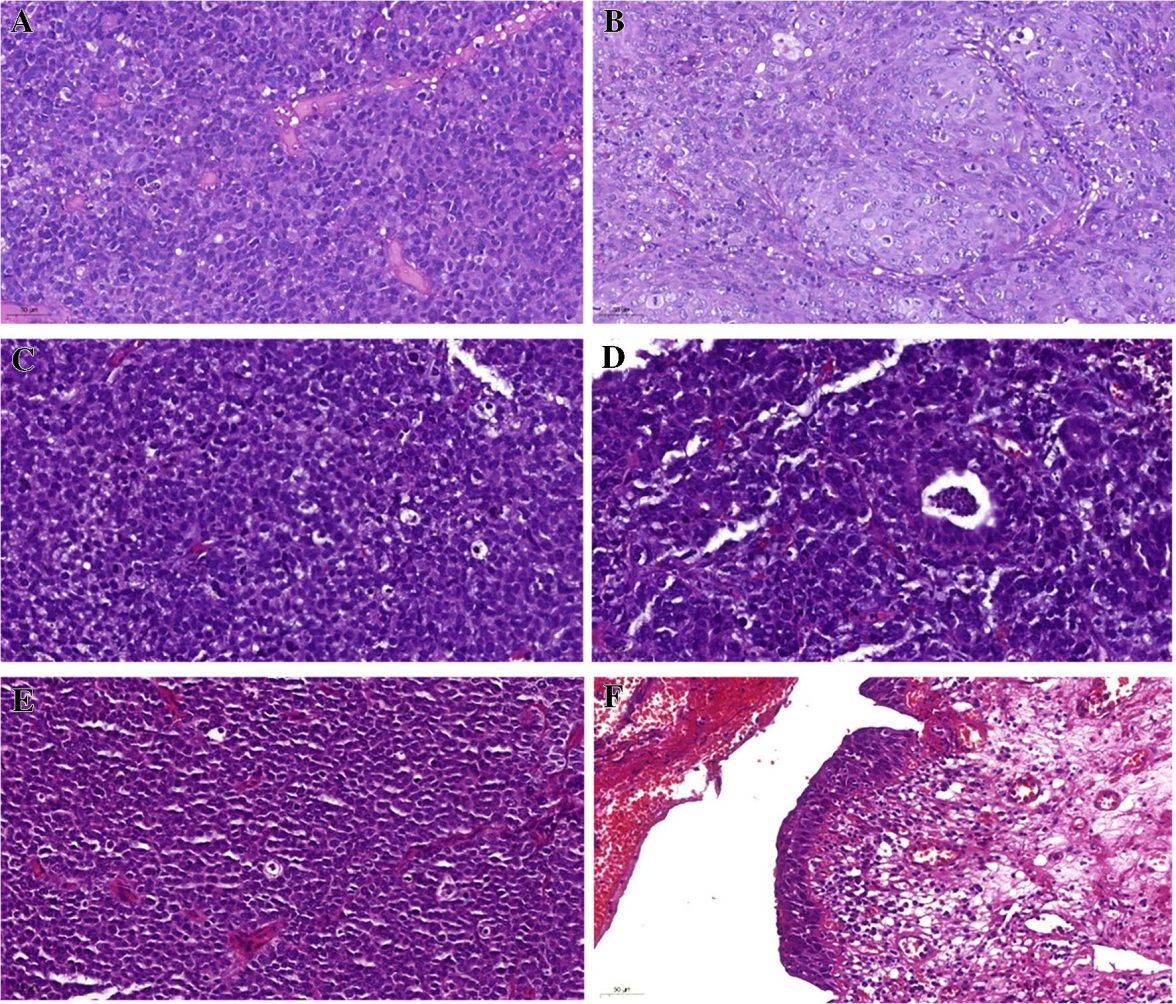
Representative images of small cell neuroendocrine carcinoma (SCNEC) of the urinary bladder: SCNEC Case 23 presenting with small cell neuroendocrine (**A**) and urothelial (**B**) components. SCNEC Case 8 (**C**) was combined with focal glandular differentiation (**D**). SCNEC (**E**) component as well as carcinoma in situ (**F**) are shown for SCNEC Case 4. All images 200 × magnification

Table 1 summarizes the clinicopathological characteristics of the cohort. Ten (43.5%) out of 23 SCNEC cases affected females. The median age at diagnosis was 67 years (range 42 to 87 years). SCNEC histologically presented with small blue round cells with high-grade morphology disposed into solid sheets and nests, diffusely invading the bladder wall layers. Mitotic activity was brisk. A urothelial component was detected in 10 (43.5%) out of 23 cases, represented by carcinoma in situ (*n* = 1), conventional invasive urothelial carcinoma component (*n* = 6) and one case each with glandular, squamous, or sarcomatoid differentiation. Representative images of the morphological appearance of SCNEC and the associated urothelial components are illustrated in Fig. 1.

**Table 1:** Study charactersitics of the analyzed SCNEC cohort

Cases	Sex	Age	Stage	Grade-2016	Grade-1973	Urothelial Component
**Small cell neuroendocrine carcinoma of the urinary bladder (Case 14 was revised to RMS due to uniform expression of desmin+myogenin)**						
1	Male	66	pT1	High-grade	G3	Conventional urothelial carcinoma
2	Male	68	pT1	High-grade	G3	Conventional urothelial carcinoma
3	Female	65	at least pT1	High-grade	G3	Conventional urothelial carcinoma
4	Female	76	pT1	High-grade	G3	Carcinoma in situ
5	Female	61	at least pT1	High-grade	G3	Not present
6	Male	61	at least pT1	High-grade	G3	Not present
7	Male	67	at least pT2	High-grade	G3	Not present
8	Female	68	pTa	High-grade	G3	Glandular
9	Female	66	at least pT1	High-grade	G3	Conventional urothelial carcinoma
10	Male	65	at least pT1	High-grade	G3	Not present
11	Female	79	pT1	High-grade	G3	Not present
12	Male	72	pT1	High-grade	G3	Not present
13	Female	55	at least pT1	High-grade	G3	Not present
14	Male	60	at least pT1	High-grade	G3	Not present (diagnosis revised to RMS due to uniform expression of desmin+myogenin)
15	Male	78	pT1	High-grade	G3	Not present
16	Female	65	at least pT1	High-grade	G3	Not present
17	Male	70	at least pT1	High-grade	G3	Not present
18	Female	42	at least pT1	High-grade	G3	Not present
19	Male	80	at least pT1	High-grade	G3	Conventional urothelial carcinoma
20	Male	82		High-grade	G3	Not present
21	Female	87	at least pT1	High-grade	G3	Squamous
22	Male	79	at least pT1	High-grade	G3	Sarcomatoid
23	Male	83	at least pT1	High-grade	G3	Conventional urothelial carcinoma
**Rhabdomyosarcoma cases of the urinary bladder**						
1	Male	69				
2	Male	72				
3	Female	68

### Immunohistochemical analysis of neuroendocrine and rhabdomyoblastic features in SCNEC

All SCNEC cases strongly expressed at least one neuroendocrine marker (mostly synaptophysin; 20/21). Variable chromogranin A expression was noted in 7 of 19 cases (moderate/strong in 6 and weak in one case). Diffuse (100%) expression of desmin and myogenin was detected in one case (Case 14), consistent with a revised diagnosis to solid RMS (Table 2). All other cases have not demonstrated any rhabdomyogenic marker expression.

**Table 2:** Results of immunohistochemical markers and expression values of both cohorts

Case	Neuroendocrine markers		Myogenic markers			
	Synaptophysin	Chromogranin A	Myogenin		Desmin	
	Intensity	Intensity	Intensity	Percentage	Intensity	Percentage
**Small cell neuroendocrine carcinoma of the bladder**						
1	Not available	Not available	No expression		No expression	
2	Not available	Not available	No expression		No expression	
3	Strong	No expression	No expression		No expression	
4	Strong	Moderate	No expression		No expression	
5	Strong	No expression	No expression		No expression	
6	Strong	No expression	No expression		No expression	
7	Strong	No expression	No expression		No expression	
8	Strong	No expression	No expression		No expression	
9	Strong	No expression	No expression		No expression	
10	Strong	Strong	No expression		No expression	
11	Strong	Weak	No expression		No expression	
12	Strong	No expression	No expression		No expression	
13	Strong	Moderate	No expression		No expression	
14	Strong	No expression	Strong	100	Strong	100
15	No expression	No expression	No expression		No expression	
16	Strong	Moderate	No expression		No expression	
17	Strong	No expression	No expression		No expression	
18	Strong	Strong	No expression		No expression	
19	Strong	No expression	No expression		No expression	
20	Weak	No expression	No expression		No expression	
21	Strong	Not available	No expression		No expression	
22	Strong	Not available	No expression		No expression	
23	Strong	Moderate	No expression		No expression	
**Rhabdomyosarcomas of the bladder**						
1	Strong		Strong	70	Strong	80
2	No expression	No expression	Strong	100	Strong	100
3	No expression	No expression	Strong	100	Strong	100

### TERT promoter gene analysis of the SCNEC cohort

Excluding the one case that was revised to RMS, 16 of the 22 genuine SCNEC cases (68%) harbored a hotspot mutation of the *TERT* promoter gene (Table 3). Almost all mutations except one occurred 124 base pairs away from the *TERT* gene. Figure 3 shows representative images of the sequence results.

**Table 3:** Results of TERT promoter mutation analysis of both cohorts

Case	*TERT* promoter mutation analysis			
	− 57	− 124	− 146	Mutational status
**Small cell neuroendocrine carcinoma of the bladder**				
1	Wild type	Wild type	Wild type	Wild type
2	Not available	G > A	Wild type	Mutated
3	Wild type	G > A	Wild type	Mutated
4	Wild type	G > A	Wild type	Mutated
5	Wild type	G > A	Wild type	Mutated
6	Wild type	G > A	Wild type	Mutated
7	Not available	G > A	Wild type	Mutated
8	Wild type	G > A	Wild type	Mutated
9	Not available	Not available	Not available	Not available
10	Wild type	Wild type	Wild type	Wild type
11	Not available	Wild type	G > A	Mutated
12	Not available	G > A	Wild type	Mutated
13	Wild type	G > A	Wild type	Mutated
14	Wild type	G > A	Wild type	Mutated
15	Wild type	G > A	Wild type	Mutated
16	Wild type	G > A	Wild type	Mutated
17	Wild type	G > A	Wild type	Mutated
18	Wild type	Wild type	Wild type	Wild type
19	Wild type	Wild type	Wild type	Wild type
20	Wild type	G > A	Wild type	Mutated
21	Wild type	Wild type	Wild type	Wild type
22	Wild type	G > A	Wild type	Mutated
23	Wild type	Wild type	Wild type	Wild type
**Rhabdomyosarcomas of the bladder**				
1	Not available	Not available	Not available	Not available
2	Wild type	G > A	Wild type	Mutated
3	Wild type	Wild type	Wild type	Wild type

### Clinicopathological characteristics and histological review of the RMS cases

**Fig. 2:**
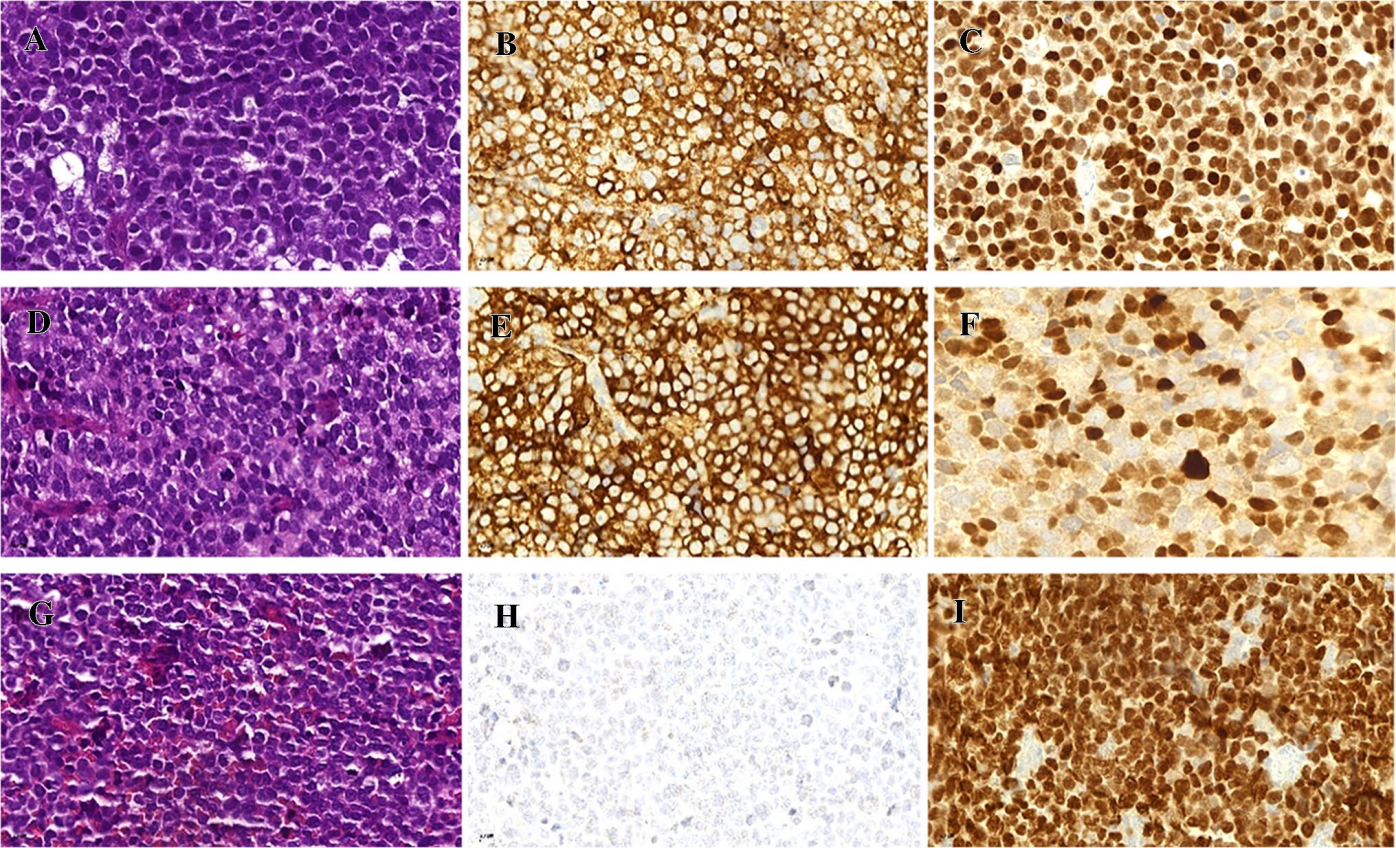
Representative images of the histology (**A**, **D**, and **G**) and immunohistochemical expression of synaptophysin (**B**, **E**, and **H**) and myogenin (**C**, **F**, and **I**) in RMS cases (upper row: SCNEC 14 that was revised to RMS; middle row: RMS Case 1; lower row: RMS Case 2); All images 400 × magnification

Table 1 summarizes the clinicopathological characteristics of the cases (including the revised SCNEC Case 14). The RMS affected three males and one female aged 60 to 72 years (median, 68). All tumors showed diffuse solid sheets of monotonous small round cells with variable dyscohesive pseudoalveolar arrangements and brisk mitotic and apoptotic activity, closely recapitulating solid-alveolar RMS of other sites. No true rhabdomyoblastic cells were seen on H&E-stained slides (Fig. 1B). A urothelial carcinoma component was lacking in all cases. Immunohistochemistry showed homogeneous expression of desmin and myogenin in 70% to 100% of cells; two cases demonstrated a strong synaptophysin expression. Representative images of the hsitology of RMS, and the different expression levels of the used immunohistochemical markers are shown in Fig. 2.

### RNA fusion and TERT promoter findings

**Fig. 3:**
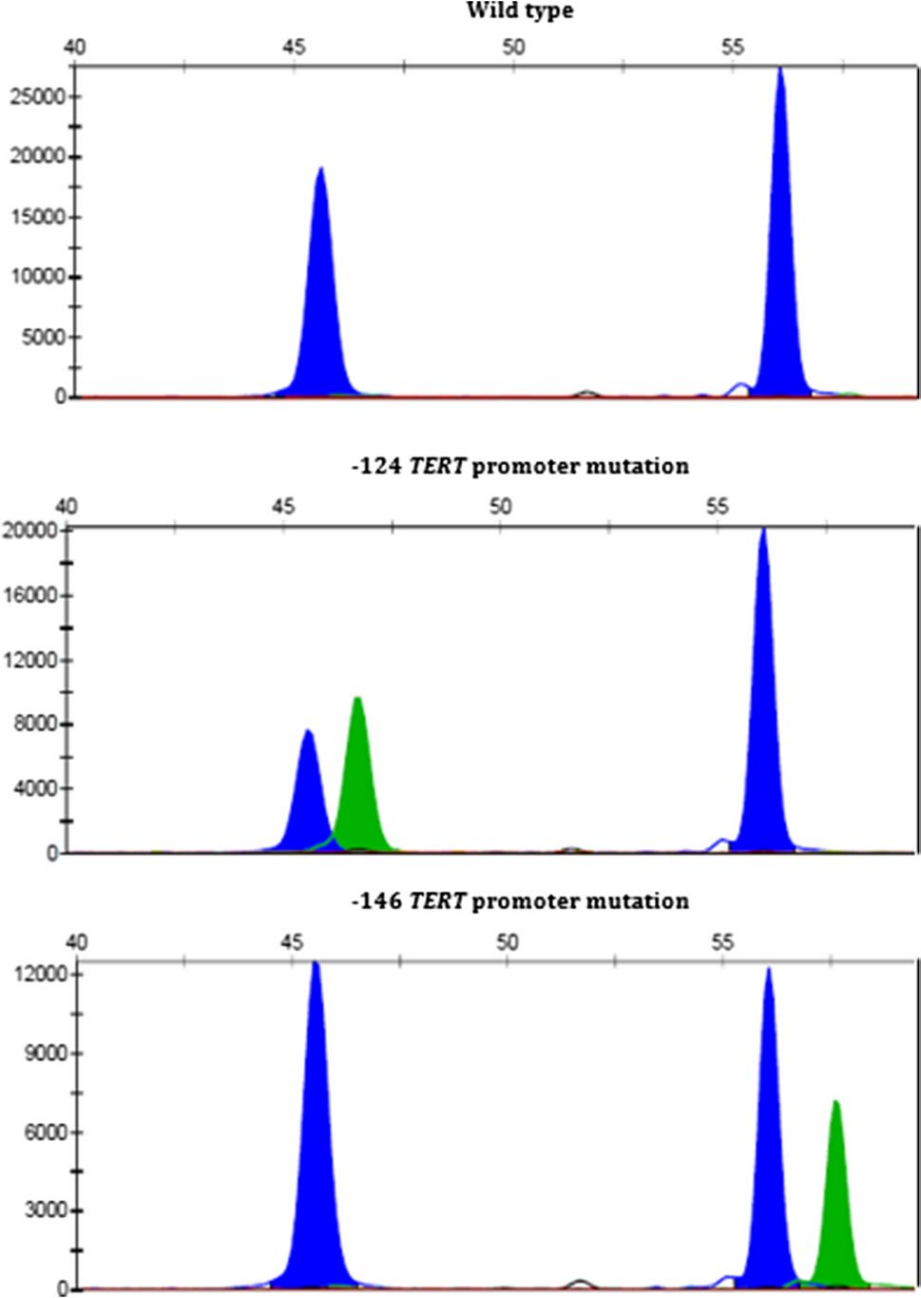
**A** Representative examples of the SNaPshot analysis of the *TERT* promoter hot spot mutations. Shown are Case 18 (upper), Case 12 (middle), and Case 11 (lower). The *X*-axis represents base pair length of the DNA fragment, and the *Y*-axis shows the intensity of fluorescence signal of the labeled nucleotide. The blue peak for each promoter mutations corresponds to a guanine and the green peak an adenine

As a surrogate marker of solid alveolar RMS, we tested the three RMS cases and the 4th case that was revised from SCNEC to RMS for gene fusions known to be frequently encountered in RMS including *FOXO1* and other fusions. None of the four cases showed a *FOXO1* fusion or fusions involving any of the 507 gene included in the TruSight RNA Fusion Panel used. Notably, this panel includes also *FUS*, *EWSR1*, *FOXO4*, *NCOA1*, *NCOA2*, *PAX3*, *VGLL3*, and *FGFR1* and is hence able to detect nearly all fusions known to be involved in adult RMS of different types. RMS Case 2 showed a LOC493754-AUTS2 fusion of unknown significance. Altogether, two of three (67%) assessable RMS cases showed a − 124 hot spot *TERT* promoter mutation (Fig. 3).

## Discussion

Significant morphological and occasionally immunophenotypic overlap is observed between SCNEC and poorly differentiated RMS originating in different organs. However, in routine practice, this overlap has no significant diagnostic impact given that the anatomic sites affected by these two entities differ significantly in most cases. Additionally, most of alveolar RMS affect children and adolescents, whereas neuroendocrine carcinomas are unexpected or do not occur among this age group, limiting the differential diagnosis. However, topographic overlap between SCNEC and poorly differentiated RMS presenting in adults is noted in a few organs, in particular, in epithelial-lined visceral organs including the sinonasal cavities and the urinary bladder. Several studies have pointed out the frequent expression of pankeratin and neuroendocrine markers in alveolar RMS [12–14]. To date, the exact cell of origin of RMS originating within these epithelial-lined organs remained elusive. Likewise, the histogenetic relationship, if any, between these poorly differentiated RMS and SCNEC has not been sufficiently explored.

After the lung, the urinary bladder is the second most common site of origin of SCNEC. In concordance with the literature, SCNEC predominately occurs in older patients with a median age at diagnosis of 67.5 years in this study. However, in contrast to its pulmonary counterpart, SCNEC of the bladder is associated with other histologic subtype/s in around 40–50% of the cases [15]. In our current series, we observed a comparable frequency of urothelial carcinoma and variants as a component in 43.5% of SCNEC.

RMS of the urinary bladder is rare, primarily occurring in children and adolescents. There are few adult cases reported in the literature [16]. While most pediatric cases correspond to the embryonal subtype of RMS, bladder RMS in adults and elderly is predominantly poorly differentiated with small cell pattern, recapitulating solid alveolar RMS [7]. Their morphology overlaps significantly with SCNEC, frequently representing pitfalls or diagnostic challenge. The issue is further complicated by frequent variable immunoreactivity of RMS cells with low molecular weight keratins and neuroendocrine markers including CD56, synaptophysin, and, less frequently, chromogranin A. In many cases, the exact diagnosis of SCNEC versus solid small cell RMS of the urinary bladder might be arbitrary and only inclusion of desmin and myogenin and the presence or absence of a urothelial component might facilitate distinction.

Rhabdomyoblastic differentiation of variable extent has been reported in high-grade NEC of different organs [6]. Although considered rare, this phenomenon might be under-diagnosed given that rhabdomyogenic markers are only applied to cases with ambiguous diagnosis or uncertain differentiation and not in clear-cut SCNEC cases. Indeed, in this study, we observed diffuse expression of desmin and myogenin in one case originally reported as SCNEC which justified revising diagnosis to RMS. On the other hand, one of our original RMS cases also presented a high expression of neuroendocrine markers. These findings highlight close phenotypic overlap between SCNEC and RMS in the bladder.

A main characteristic of urinary bladder cancer are mutations in the *TERT* promoter gene. Hotspot mutations of this promoter were detected in around 70% of bladder cancer cases and were not associated with clinical or pathological parameters [17]. In a recent publication on 132 SCNEC of the bladder, *TERT* promoter mutations were detected in 68% of cases [18]. We herein report similar frequency in our cohort of 22 SCNEC (68%). In the context of a bladder neoplasm, detection of *TERT* promoter mutation has emerged as a surrogate marker for urothelial origin. As reported by Priemer et al., the *TERT* promoter mutational status can differentiate SCNEC of the bladder from those of prostatic origin [19].

In our current study, we found significant morphological and immunophenotypic overlap between SCNEC and RMS of the bladder. The only distinguishing diagnostic differences of RMS are the lack of a urothelial component and the presence of homogeneous rhabdomyoblastic immunophenotype. Our findings suggest a continuum of rhabdomyoblastic differentiation in both entities ranging from virtually absent in classical SCNEC on one end of the spectrum to being the sole pattern in tumors classified as RMS on the opposite end of the spectrum with possible intermediate variants in between, although we have not observed cases with focal or partial rhabdomyoblastic differentiation, but this might be the result of sampling errors. Consistent with this view, none of the four RMS cases tested with targeted RNA sequencing showed any of the gene fusions expected in genuine alveolar-type RMS. The common origin of SCNEC and RMS in the bladder was further strengthened by the high comparable frequency of *TERT* promoter mutations identified in both entities in our study (68% of SCNEC and 67% of RMS cases, respectively). According to the current literature, *TERT* promoter mutations are very rare in RMS and were detected in approximately 1.4% of RMS cases not stratified by the organ of origin [20]. On the other hand, > 80% of all alveolar RMS harbor a distinct balanced *FOXO1* translocation, which is a characteristic and disease-defining genetic marker [8].

Thompson et al. [14] analyzed 52 alveolar RMS of the sinonasal tract in adults aged ≥ 18 years (mean age, 43) and detected low-molecular weight keratins overall in 54% of cases (CAM5.2 in 50% and AE1/AE3 in 36%). Moreover, the neuroendocrine markers CD56 (100%), synaptophysin (35%), and chromogranin (13%) were frequently expressed. However, despite this significant phenotypic overlap with sinonasal SCNEC, the histogenesis and molecular pathogenesis of adult RMS in these epithelial-lined organs seem quite distinct and site-specific. Notably, FOXO1 rearrangements have been detected by PCR studies in 81% of sinonasal RMS cases with PAX3 as fusion partner in 72.7% of cases [14]. On the contrary, none of our 4 RMS cases and none of four unclassified bladder RMSs in adults studied by Gupta et al. revealed a FOXO1 or other RMS-associated fusion [21]. A fusion involving PPP1R12A (fused to LIN7A or PTPRQ) was detected by Gupta et al. in two RMS cases (one reported as sarcomatoid!). However, these gene fusions have not been reported before, and it is not clear if they drive oncogenesis or represent passenger events. Admittedly, the fusion partners detected by Gupta et al. in two tumors are not included in the TruSight Illumina Panel we used in this study, so the relevance of this fusion needs to be verified in larger future studies.

Treatment of poorly differentiated RMS (including solid alveolar subtypes), in adults typically involves a combination of agents such as cyclophosphamide, vincristine, and doxorubicin [22]. In contrast, standard chemotherapy for SCNEC of the urinary bladder often utilizes platinum-based chemotherapy and etoposide [23]. Accordingly, separating these two disease entities has clear therapeutic relevance. However, distinguishing these two aggressive phenotypic diseases in routine practice is challenging and is significantly influenced by extent of sampling. Thorough sampling and careful histological evaluation to detect minor microscopic urothelial foci (in situ or invasive) is the most reliable clue for verifying a urothelial origin. However, in limited biopsies and in cases with uniform rhabdomyoblastic differentiation, only inclusion of desmin and myogenin in the immunohistochemical panel of any potential SCNEC would facilitate this distinction. Our study indicates that TERT promoter mutations are identified in 67% of assessable RMS cases, representing a potential surrogate for a urothelial origin. Enhanced recognition of RMS cases should help to confirm our hypothesis, and if confirmed, then to address the central question, whether the approach to treat adult RMS of the urinary bladder needs to be revised. Moreover, targeting the urothelial origin through additional modified therapeutic approaches could potentially lead to improved response rates.

In summary, our study confirms the reported significant morphological and immunophenotypic overlap between SCNEC and poorly differentiated RMS of the urinary bladder. We herein add molecular overlap with similar frequency of TERT promotor mutations in SCNEC (68%) and RMS (67%) in our study. The presence of *TERT* promoter mutations and lack of *FOXO1* and other RMS gene fusions in all tested RMS cases are in line with a urothelial origin of most if not all RMS of the bladder in adults. Urinary bladder small round cell RMS in adults probably originates via monomorphic rhabdomyoblastic transdifferentiation in SCNEC and are likely distinct from genuine alveolar RMS in other organs/ soft tissue and bone sites. Our study is however limited by the low number of RMS cases due to rarity of this disease. Accordingly, extended analysis on more RMS cases is needed to further consolidate this notion.

## Data Availability

The datasets generated during and/or analyzed during the current study are not publicly available, but are available from the corresponding author on reasonable request.

## Declarations

This study was conducted in accordance with the Declaration of Helsinki and the protocol approved by the Ethics Committee of the Friedrich-Alexander University Erlangen-Nürnberg (No. 329_16B).
